# ﻿Two new species and new records of Otocepheidae (Acari, Oribatida) from Yunnan, Southwest China

**DOI:** 10.3897/zookeys.1073.75583

**Published:** 2021-11-30

**Authors:** Lihao Zheng, Jun Chen

**Affiliations:** 1 Guang’an Vacational and Technical College, Guang’an, China Institute of Zoology, Chinese Academy of Sciences Beijing China; 2 Key Laboratory of Zoological Systematics and Evolution, Institute of Zoology, Chinese Academy of Sciences, Beijing 100101, China Guang’an Vacational and Technical College Guang’an China; 3 College of Life Sciences, University of Chinese Academy of Sciences, Beijing, 100049, China University of Chinese Academy of Sciences Beijing China

**Keywords:** *Basiceramerus*, China, Eurostocepheus (Eurostocepheus), oribatid mites, taxonomy

## Abstract

This work includes taxonomic data on four species of oribatid mites of the family Otocepheidae from Yunnan, China. Two new species of the genera *Basiceramerus* and *Eurostocepheus* are described, respectively. *Basiceramerusovatus***sp. nov.** differs from *B.bangladeshensis* Corpuz-Raros & Gruèzo, 2008 by the wavy marginalis passing the base of the adanal setae, prodorsal condyles well separated from median ones, a ventral ridge present, and the anal plate foveolate; it differs from *B.igorotus* Corpuz-Raros & Gruèzo, 2011 from Vietnam by the wavy marginalis passing the base of the adanal setae, a connected tutorium and lamelliform expansion, a ventral ridge present, a smooth genital plate, and seta *an*_2_ located close to the median margin of the anal opening; it differs from *B.igorotus* from the Philippines by the lamellar setae inserted behind the tip of the lamella, separated prodorsal condyles, lyrifissure *im* posterior to *gla*, genital plate smooth, anal plate foveolate, and the wavy marginalis passing the base of the adanal setae. Eurostocepheus (Eurostocepheus) sinutus**sp. nov.** differs from other known species of this genus by having a ventral groove between the genital aperture and the ventral ridge, eight pairs of notogastral setae, and distinctly shorter and thinner notogastral setae *p*_1_, *p*_2_, *p*_3_, *h*_3_. Eurostocepheus (Eurostocepheus) aquilinus Aoki, 1965 and E. (E.) mahunkai Mondal & Kundu, 1999 are reported for the first time from China.

## ﻿Introduction

During identification of the oribatid mite material collected from Yunnan, Southwest China, we found four otocepheid species; among them two species are new to science belonging to the genera *Basiceramerus* and *Eurostocepheus*, and the others, Eurostocepheus (Eurostocepheus) aquilinus Aoki, 1965 and E. (E.) mahunkai Mondal & Kundu, 1999, are new records for China.

*Basiceramerus* was proposed by Corpuz-Raros with *Basiceramerusupelbensis* Corpuz-Raros, 1979 as the type species. Currently, the genus comprises six species, which are distributed in the subtropics of Asia: the Philippines, Bangladesh and Vietnam (Subías 2004, online version 2021). Before the present study, this genus had not been reported from China. The species herein described follows the generic characters (based on data from Corpuz-Raros 1979; Corpuz-Raros and Gruèzo 2008): fused median notogastral condyles present, apodemata II and apodemata *sj* long, 4 pairs genital, 1 pair aggenital, 2 pairs anal, 3 pairs adanal setae present, and leg setae *u* setiform (L-type) on tarsi I, thorn-like (S-type) on tarsi II–IV.

*Eurostocepheus* Aoki, 1965, which is distinguished from other genera of Otocepheidae mainly by its disproportionately dilated pedotectum II and conspicuously developed costula, comprises two subgenera: Eurostocepheus (Eurostocepheus) Aoki, 1965 and Eurostocepheus (Cerostocepheus) Mahunka, 1973. The main subgeneric difference lies in the number of genital setae, either 4 or 5 pairs respectively. Nine species of this genus, all from the Oriental region, were hitherto reported (Subías 2004, online version updated in 2021); among them only one species, Eurostocepheus (E.) heterotrichus Wen, 1999, has been recorded in China (Wen 1999; Chen, Liu and Wang 2010). A revised generic diagnosis and an identification key to known subgenera and species of this genus were given by Ermilov and Starý (2017).

In the following study, the two new species *Basiceramerusovatus* sp. nov., Eurostocepheus (Eurostocepheus) sinutus sp. nov., are described and illustrated based on adults, and expanded descriptions and illustrations of E. (E.) aquilinus and E. (E.) mahunkai based in part on new information are provided.

## ﻿Materials and methods

Specimens were mounted in lactic acid on temporary cavity slides for measurement and illustration. The body length was measured in lateral view, from the tip of the rostrum to the posterior edge of the ventral plate. Notogastral width refers to the maximum width in dorsal aspect. Lengths of body setae were measured in lateral aspect. All body measurements are presented in micrometers. Formulae for leg setation are given in parentheses according to the sequence trochanter-femur-genu-tibia-tarsus (famulus included). Formulae for leg solenidia are given in square brackets according to the sequence genu-tibia-tarsus.

General terminology used in this paper follows that of Grandjean (1934), Ermilov and Starý (2017), Norton (1977), Norton and Behan-Pelletier (2009).

### ﻿Abbreviations and notations

**Prodorsum**: *ro*, *le*, *in*, *bs*, *ex*–rostral, lamellar, interlamellar, bothridial and exobothridial setae, respectively; *cos*–costula; *tu*–tutorium; *spa.l*–lamelliform expansion; *tbd*, *tbv*–dorsal and ventral bothridial plate, respectively; *cpm*, *cpl*–medial and lateral prodorsal condyles, respectively.

**Notogaster**: *c*, *la*, *lm*, *lp*, *h*-row, *p*-row–notogastral setae; *cnm*, *cnl*–medial and lateral notogastral condyles, respectively; *vm*–vitta marginalis; *ia*, *im*, *ip*–anterior, middle, posterior lyrifissures, respectively; *ih*, *ips*–same, associated with setal rows *h* and *p*, respectively; *gla*–opisthonotal gland opening.

**Coxisternum and lateral podosoma**: *1a*, *1b*, *1c*, *2a*, *3a*, *3b*, *3c*, *4a*, *4b*, *4c*–setae of epimeres I–IV; *met*–mentotectum; *st*–sternal apodeme; *ap1*, *ap2*, *ap sj*–apodeme I, II, sejugal, respectively; *Pd I*, *Pd II*–pedotectum I, II respectively; *spd*–sub pedotectum; *fep*–epimeral foramen; *dis*–discidium; *opp*–postpodosomal ornamentation.

**Anogenital region**: *g*, *ag*, *an*, *ad*–genital, aggenital, anal and adanal setae, respectively; *vr*–ventral ridge; *iag*, *iad*–aggenital, adanal lyrifissure respectively.

**Gnathosoma**: *a*, *m*–anterior, middle seta of gena; *h*–hypostomal seta of mentum; *v*, *l*, *d*, *cm*, *acm*, *ul*, *su*, *vt*, *lt*, *sup*, *inf*–palp setae; **ω**–palp tarsal solenidion; *ep*–postpalpal seta; *cha*, *chb*–cheliceral setae; *cht*–tooth on dorsal chelicerae; *rbr*–rutellar brush; Tg–Trägårdh’s organ.

**Legs**: σ, φ, ω–solenidia of genu, tibia and tarsus, respectively; *ɛ*–famulus of tarsus I; *d*, *l*, *v*–dorsal, lateral, ventral setae, respectively; *ev*, *bv*–basal trochanteral setae; *ft*, *tc*, *it*, *p*, *u*, *a*, *s*, *pv*–tarsal setae; Tr, Fe, Ge, Ti, Ta–trochanter, femur, genu, tibia, tarsus of legs, respectively.

## ﻿Taxonomy

### ﻿ Basiceramerus ovatus sp. nov.

Taxon classificationAnimaliaSarcoptiformesOtocepheidae

D8FDD5A0-B36F-5177-98EA-DBF8C73AFAB3

http://zoobank.org/E8AFB948-9A53-47EF-9A1A-CFF080297747

**Figure 1.**
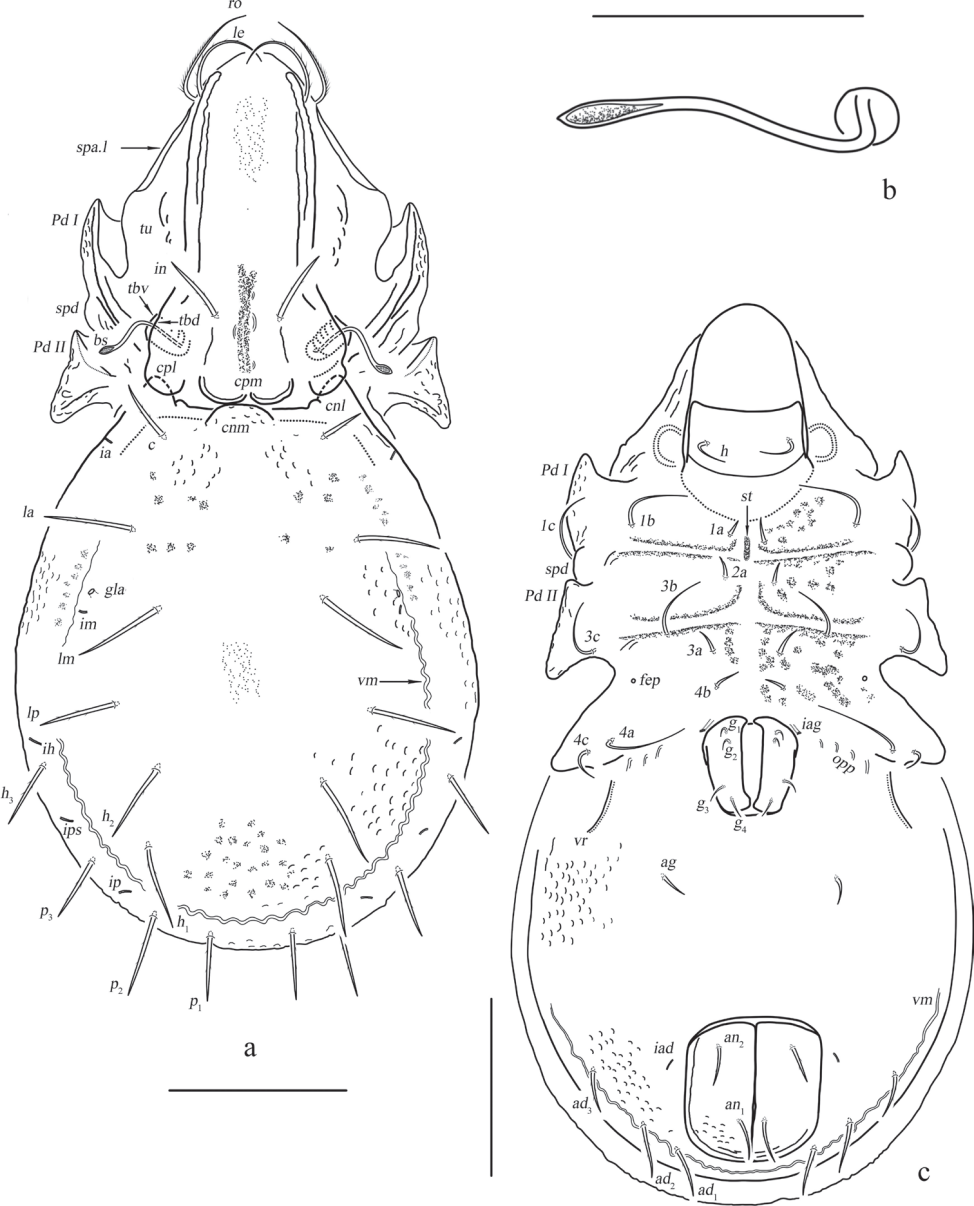
*Basiceramerusovatus* sp. nov. adult: **a** dorsal view (legs not illustrated) **b** bothridial seta **c** ventral view (legs not illustrated). Abbreviations and notations explained in text. Scale bars: 200 μm (**a, c**); 100 μm (**b**).

**Figure 2.**
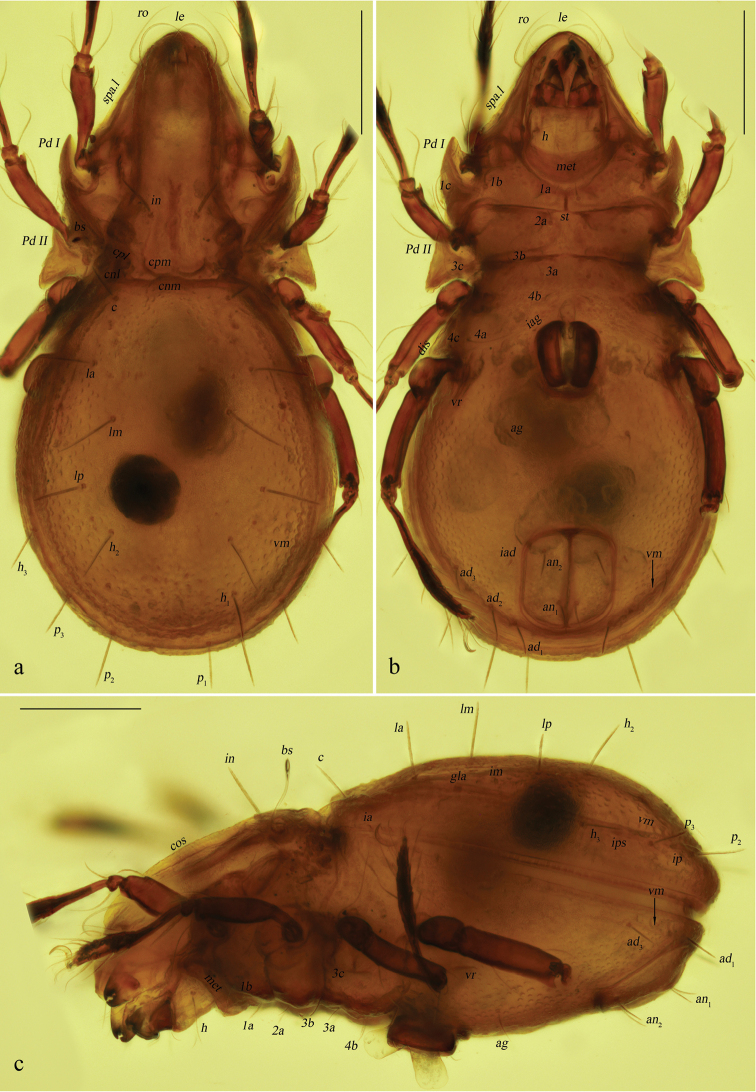
*Basiceramerusovatus* sp. nov., adult, microscope images: **a** dorsal view **b** ventral view **c** lateral view. Abbreviations and notations explained in text. Scale bars: 200 μm (**a–c**).

**Figure 3.**
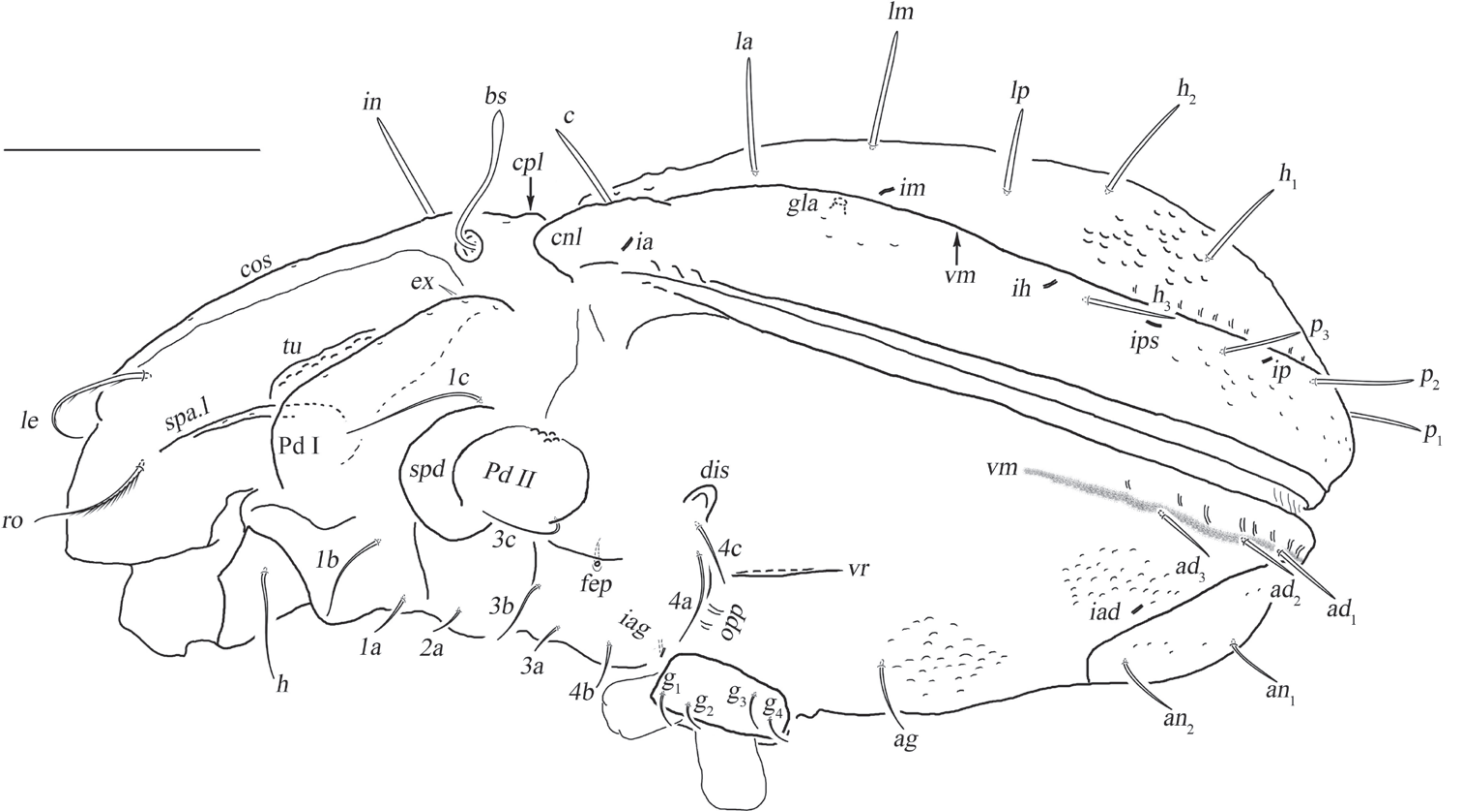
*Basiceramerusovatus* sp. nov. adult: lateral view (legs not illustrated). Abbreviations and notations explained in text. Scale bar: 200 μm.

**Figure 4.**
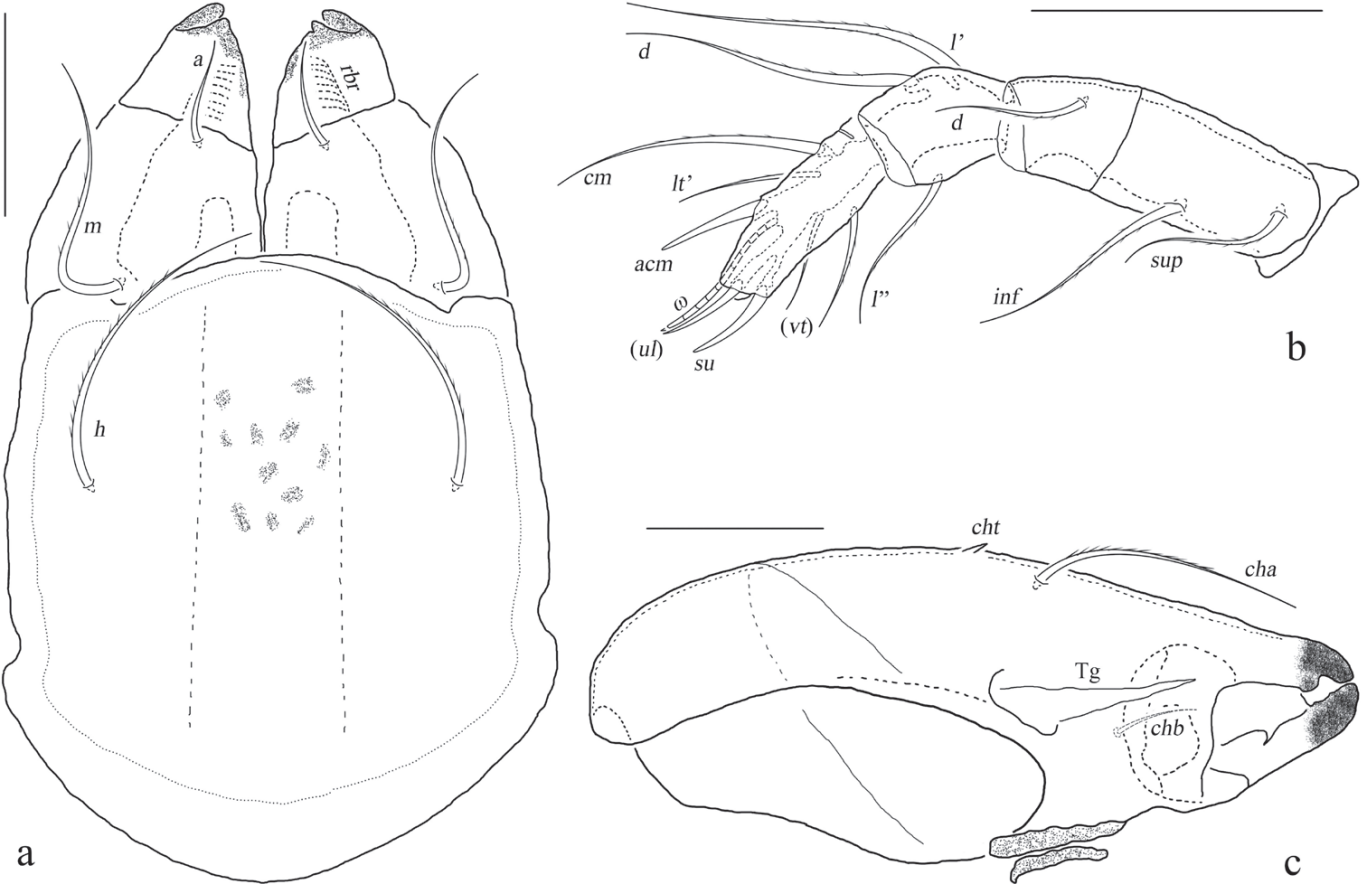
*Basiceramerusovatus* sp. nov. adult: **a** subcapitulum, ventral view **b** left palp, abaxial view **c** left chelicera, adaxial view. Abbreviations and notations explained in text. Scale bars: 50 μm.

**Figure 5.**
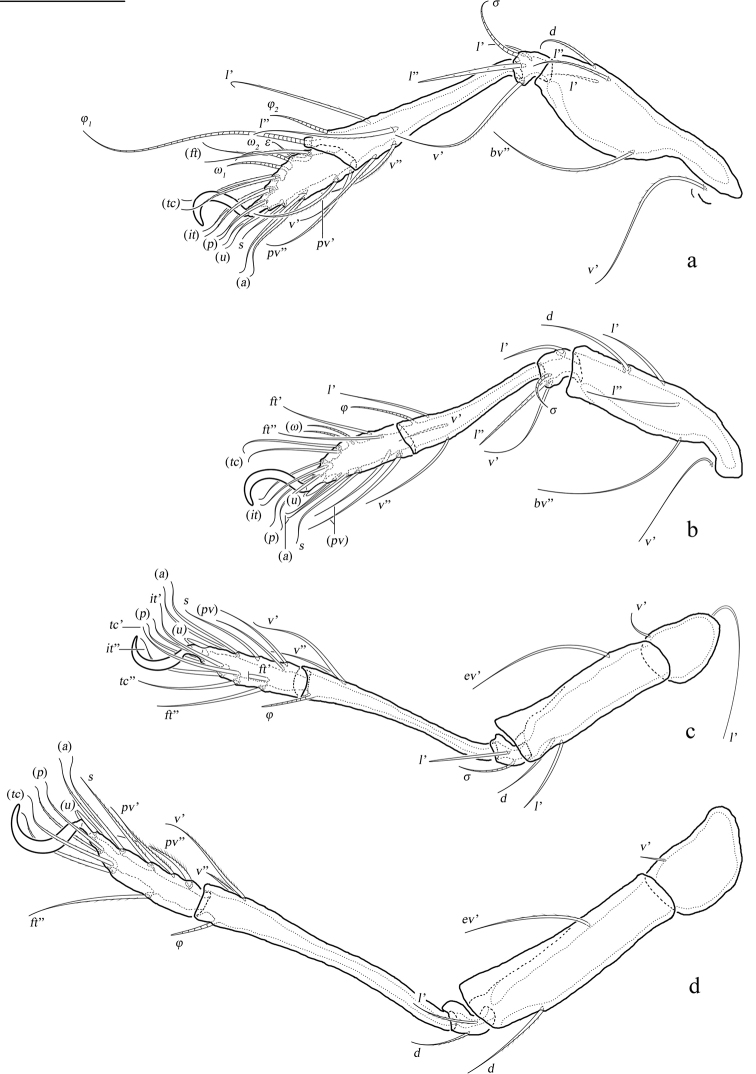
*Basiceramerusovatus* sp. nov., adult: **a–d** leg I–IV, left, antiaxial view. Abbreviations and notations explained in text. Scale bars: 100 μm (**a–d**).

Figures 1 , 2 , 3 , 4 , 5

#### Diagnosis.

Body size (*N* = 4): 990–1360 × 540–650. Two pairs of prodorsal condyles present, similar in shape, broadly rounded, median prodorsal condyles close to each other but not fused. Lateral notogastral condyles triangular, with a tiny convex at bottom. One median notogastral condyle, rounded. Ten pairs of notogastral setae. Vitta marginalis distinct. A wavy marginalis, like vitta marginalis, passing the base of adanal setae, ended at level of anterior margin of anal opening.

#### Description.

***Measurements*.** Body length: 1020 (holotype, male), 990–1360 (paratypes, two males and one female), body width: 540 (holotype, male), 540–650 (paratypes, two males and one female). Setae length and mutual distance (holotype, male): *ro* 120, *le* 140, *bs* 130, *in* 110, *ex* 20; *c*, *la*, *lm*, *lp*, *h*_1_, *h*_2_, *h*_3_, *p*_1_, *p*_2_, *p*_3_ range 80–100; *c*–*c* 370, *la*–*la* 430, *lm*–*lm* 470, *lp*–*lp* 470.

***Integument*.** Body color light brownish. Surface of notogaster foveolate.

***Prodorsum*.** Rostrum rounded. Rostral setae moderately curved inward, densely barbed outside. Lamellar setae inserted behind tip of lamella, curved inward, roughened outside. Interlamellar setae barbed and setiform, a pair of longitudinal wrinkles extending from its bottom backward to outer margin of median prodorsal condyles. Exobothridial setae short. Bothridial seta with a fusiform head. Tutorium developed, almost touching lamelliform expansion. Lamelliform expansion pointing to bottom of seta *ro.* Bothridium opening laterally, dorsal bothridial plate nearly straight, ventral bothridial plate broadly rounded in dorsal view. Two pairs of prodorsal condyles present, similar in shape, broadly rounded, median prodorsal condyles close to each other but not fused. Mutual distance between ventral bothridial plates nearly equal with that between lateral prodorsal condyles.

***Notogaster*.** L/W of notogaster about 1.3. Lateral notogastral condyles triangular, with a tiny convex at bottom. One median notogastral condyle present, rounded. Ten pairs of notogastral setae, glabrous, setiform, nearly equal in length. Setae *lm* and lyrifissures *im* located nearly same level. All lyrifissures (*im*, *ip*, *ih*, *ips*, except *ia*) well visible in dorsal view, *ip* located between *p*_2_ and *p*_3_, *ips* between *h*_3_ and *p*_3_. Opisthonotal gland openings located anterior and very close to *im.* Vitta marginalis distinct.

***Epimeral and lateral podosomal regions*.** Apodemes II and sejugal apodeme well developed, apodemes III invisible, epimeral foramen present, pedotectum II with anterior and posterior expansions nearly equal in size. Sternal apodeme well visible. Epimeral setal formula 3-1-3-3. Seta *4a* inserted between *4b* and *4c*, and closer to *4c*. Postpodosomal ornamentation well developed.

***Anogenital region*.** Genital plates smooth. Four pairs of genital setae (mutual distances *g*_1_–*g*_1_≈*g*_2_–*g*_2_≈*g*_4_–*g*_4_<*g*_3_–*g*_3_). Aggenital lyrifissures located close and anterolateral to genital aperture. One pair of aggenital, two pairs of anal (mutual distances *an*_1_–*an*_1_<*an*_2_–*an*_2_, seta *an*_1_ located close to median margin of anal opening) and three pairs of adanal setae similar in length. Setae *ad*_3_–*ad*_3_ well below level of anterior margin of anal opening. Anal plate foveolate. Lyrifissures *iad* located in diagonal position and close to anal aperture, below level of anterior margin of anal opening. A wavy marginalis, like vitta marginalis, passing the base of adanal setae, ending beyond level of anterior margin of anal opening.

***Gnathosoma*.** Subcapitular setae barbed, flagelliform at tips. Rutellum pantelobasic, with typical dentation and rutellar brush. Chelicera chelate-dentate; with a minute denticle proximal to seta *cha*; *cha* longer than *chb*, both of them barbed; Trägårdh’s organ narrowly triangular. Palp with usual setal formula: 0-2-1-3-8 (+**ω**); setae of trochanter to tibia barbed. Tarsus with four short, blunt distal eupathidia–*acm*, *su*, (*ul*); other tarsal setae smooth or with sparse, inconspicuous barbs; base of solenidion **ω** constrainted by surface of tarsusand thus adjacent to setae *ul*’, *ul*” medioanteriorly. Postpalpal setae erect, smooth.

***Legs*.** Monodactylous. Claw of each leg strong and smooth. Formulae of leg setation and solenidia (Table 1): I (1-4-3-4-16) [1-2-2], II (1-4-3-3-15) [1-1-2], III (2-3-1-2-15) [1-1-0], IV (1-2-2-2-12) [0-1-0]. Leg setae *u* setiform (L-type) on tarsi I, thorn-like (S-type) on tarsi II–IV.

**Table 1.** Leg setation and solenidia of adult *Basiceramerusovatus* sp. nov. Roman letters refer to normal setae, Greek letters to solenidia (except *ɛ*=famulus). Single prime (’) marks setae on the anterior and double prime (”) setae on the posterior side of a given leg segment. Parentheses refer to a pair of setae. Tr – trochanter, Fe – femur, Ge – genu, Ti – Tibia, Ta – tarsus.

Leg	Tr	Fe	Ge	Ti	Ta
Ⅰ	*v*’	*d*, (*l*), *bv*”	(*l*), *v*’, *σ*	(*l*), (*v*), *φ_1_*, *φ_2_*	(*ft*), (*tc*), (*it*), (*p*), (*u*), (*a*), *s*, (*pv*), *ε*, *ω_1_*, *ω_2_*
Ⅱ	*v*’	*d*, (*l*), *bv*”	(*l*), *v*’, *σ*	*l*’, (*v*), *φ*	(*ft*), (*tc*), (*it*), (*p*), (*u*), (*a*), *s*, (*pv*), *ω_1_*, *ω_2_*
Ⅲ	*l*’, *v*’	*d*, *l*’, e*v*’	*l*’, *σ*	(*v*), *φ*	(*ft*), (*tc*), (*it*), (*p*), (*u*), (*a*), *s*, (*pv*)
Ⅳ	*v*’	*d*, e*v*’	*d*, *l*’	(*v*), *φ*	*ft*”, (*tc*), (*p*), (*u*), (*a*), *s*, (*pv*)

#### Material examined.

***Holotype***: male (in alcohol, ZLH-12-225): China, Yunnan Province, Ruili City, Nongdao Town, Nankaiba Village, 23°54'51.19"N, 97°33'58.69"E, 835 m a. s. l., in soil and debris under bush, 23 October 2012. ***Paratypes***: two females (in alcohol, ZLH-12-225): same data as holotype; one female (in alcohol, ZLH-12-259): China, Yunnan Province, Yingjiang County, Daonong Village, 24°40'2.568"N, 97°35'54.24"E, 924 m a. s. l., in soil and debris under bush, 31 October 2012. All type specimens were collected by Lihao Zheng.

#### Type deposition.

All type specimens are deposited in the collection of the Institute of Zoology, Chinese Academy of Sciences, Beijing (IZAS).

#### Etymology.

The specific name “*ovatus*” is from Latin for “egg” refers to the oval notogaster in dorsal view.

#### Remarks.

The new species is morphologically similar to *B.bangladeshensis* Corpuz-Raros & Gruèzo, 2008 from Bangladesh and *B.igorotus* Corpuz-Raros & Gruèzo, 2011 from the Philippines and Vietnam (Ermilov and Anichkin 2013) in having two median prodorsal condyles. However, the new species differs from *B.bangladeshensis* by the wavy marginalis, like vitta marginalis, passing the base of adanal setae (vs. none), prodorsal condyles well separated from the median ones (vs. all prodorsal condyles touching at base), ventral ridge present (vs. none), anal plate foveolate (vs. granulate, without foveolae). The species *B.igorotus* was reported from the Philippines and Vietnam by Corpuz-Raros and Gruèzo (2011) and Ermilov and Anichkin (2013) respectively. The latter recorded instances of intraspecific or geographical variability based on their specimens from Vietnam: body size larger and more elongate, interlamellar setae shorter, lamellar setae longer, lateral notogastral condyles narrower, medial notogastral condyles touching base of lateral notogastral condyles, genital plate smooth in the Vietnamese specimens. The new species differs from *B.igorotus* from Vietnam by the wavy marginalis passing the base of adanal setae (vs. none), tutorium and lamelliform expansion nearly touching (vs. well separated), ventral ridge present (vs. none), genital plate smooth (vs. finely striate), seta *an*_1_ located close to the median margin of the anal opening (vs. *an*_1_ well removed from median margin of anal opening); it differs from *B.igorotus* from the Philippines by the lamellar setae inserted behind the tip of the lamella (vs. lamellar setae arising outside the base of cuspis), separated prodorsal condyles (vs. prodorsal condyles all touching at base), *im* posterior to *gla* (vs. *im* anterior to *gla*), genital plate smooth (vs. finely striate), anal plate foveolate (vs. granulate), wavy marginalis, like vitta marginalis, passing the base of adanal setae (vs. none).

### ﻿ Eurostocepheus (Eurostocepheus) sinutus sp. nov.

Taxon classificationAnimaliaSarcoptiformesOtocepheidae

F75CCE25-AFB8-5658-893A-DEA5CBE9CBB2

http://zoobank.org/2500C6A8-CB6A-44BF-9C05-1B85F9F80471

Figures 6 , 7 , 8

#### Diagnosis.

Body size: 1500 × 850. Body ratio (length/width): 1.8. Body surface relatively smooth. Costula strong, a little “S” shaped curved, largest width of mutual distance anteriorly, curved inward around setae *le.* Pedotecta II disproportionately dilated, with smaller anterior parts and larger posterior parts projecting lateroposteriad. Eight pairs of notogastral setae, *c*, *la*, *lm*, *lp* setiform and slightly barbed distally, *p*_1_, *p*_2_, *p*_3_, *h*_3_ short and ciliform. Epimeral setal formula 3-1-3-3. A pair of ventral grooves between genital aperture and ventral ridge present.

**Figure 6.**
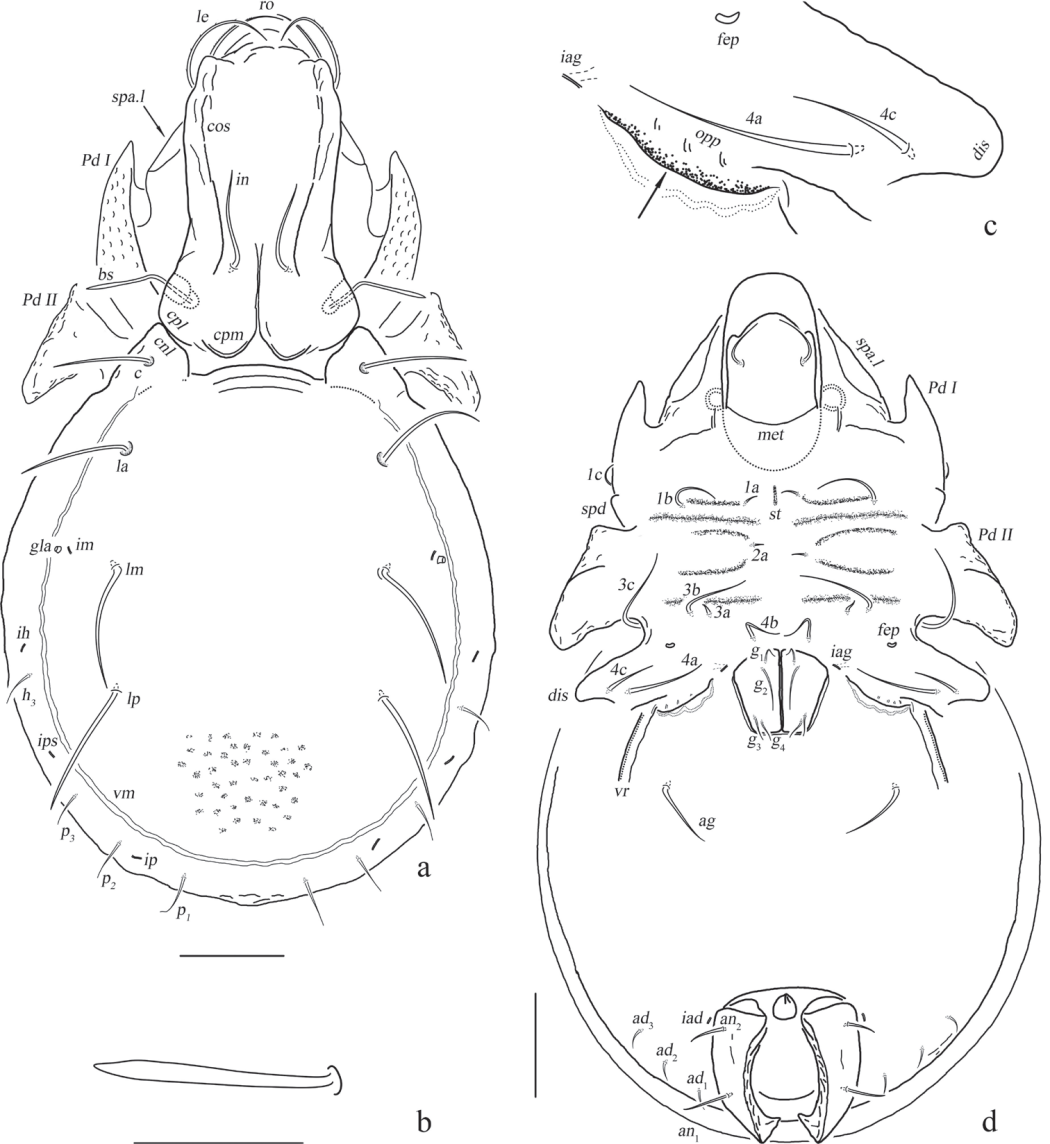
Eurostocepheus (Eurostocepheus) sinutus sp. nov., adult: **a** dorsal aspect (legs removed) **b** bothridial seta **c** epimeron IV (left, part), showing ventral groove (arrow) **d** ventral aspect (legs and mouthparts removed). Abbreviations and notations explained in text. Scale bars: 200 µm (**a, d**); 100 µm (**b**).

#### Description.

***Measurements*** (holotype, female). Body length: 1500, body width: 850. Setae length and mutual distance: *ro* 230, *le* 230, *bs* 150, *ex* 20; *c*, *la*, *lm*, *lp* range 210–250; *p*_1_, *p*_2_, *p*_3_, *h*_3_ range 40–60; *c*–*c* 370, *la*–*la* 430, *lm*–*lm* 470, *lp*–*lp* 470.

**Figure 7.**
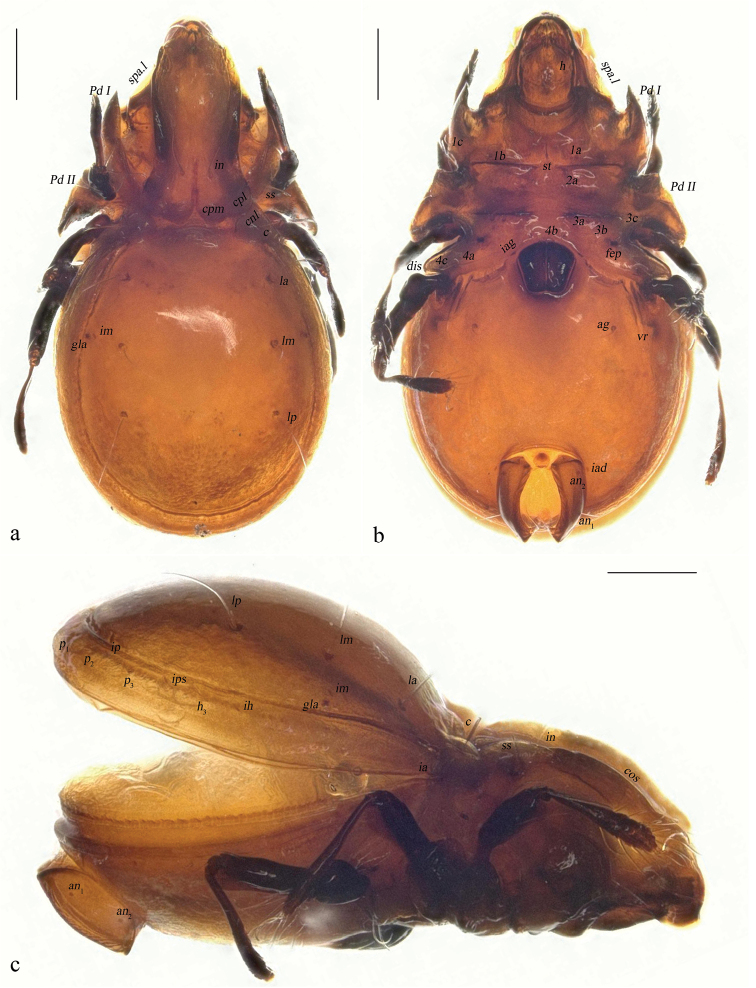
Eurostocepheus (Eurostocepheus) sinutus sp. nov., adult, microscope images: **a** dorsal view **b** ventral view **c** lateral view. Abbreviations and notations explained in text. Scale bars: 200µm.

***Integument*.** Body color dark brownish. Body surface relatively smooth.

***Prodorsum*.** Rostrum broadly rounded. Rostral setae curved inward, densely barbed outside. Lamellar setae inserted behind tip of costula, curved inward, roughened externally. Interlamellar setae slightly barbed. Bothridial setae with a long fusiform head and a curved peduncle in dorsal view. Exobothridial setae short, hardly visible in dorsal view. Costula strong, weakly “S” shaped, largest width of mutual distance anteriorly, curved inward around setae *le.* Bothridium opening laterally, dorsal bothridial plate nearly straight, ventral bothridial plate invisible in dorsal view. Tutorium developed weakly. Lamelliform expansion curved and pointing to base of seta *ro* in lateral view. Two pairs of prodorsal condyles present, lateral prodorsal condyles broadly flattened and wide, median prodorsal condyles drop-shaped.

**Figure 8.**
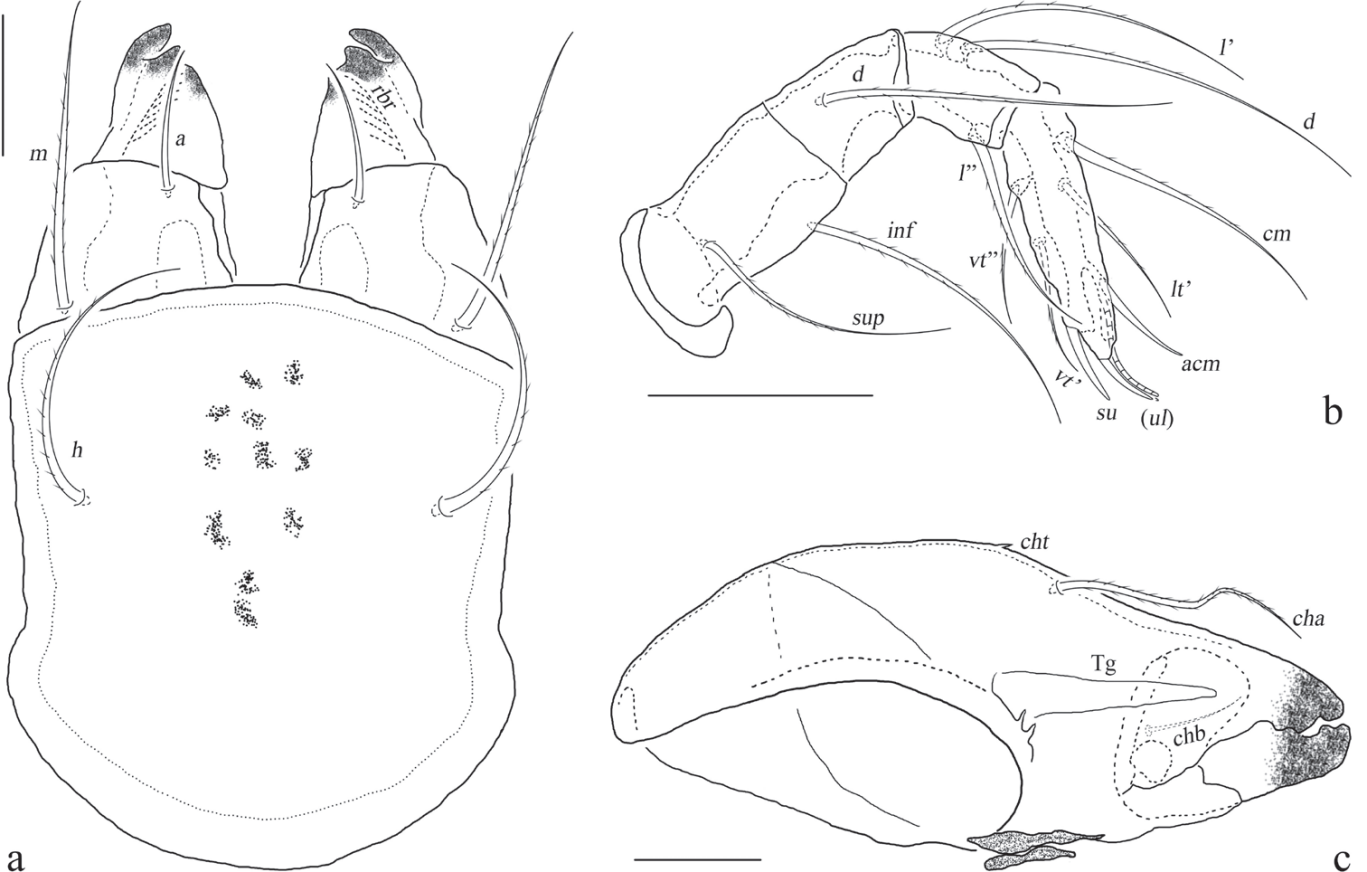
Eurostocepheus (Eurostocepheus) sinutus sp. nov., adult: **a** subcapitulum, ventral view **b** right palp, abaxial view **c** left chelicera, adaxial view. Abbreviations and notations explained in text. Scale bars: 50 μm.

***Notogaster*.** L/W of notogaster about 1.1. Surface of notogaster relatively smooth in dorsal view, without visible foveola or granules. Anterior margin of notogaster distinctly moved forward. Lateral notogastral condyles trapezoid, with triangular tip outside, which markedly anterior to medial prodorsal condyles. Median notogastral condyles absent. Eight pairs of notogastral setae, *c*, *la*, *lm*, *lp* longer than others distinctly, setiform and slightly barbed distally, *p*_1_, *p*_2_, *p*_3_, *h*_3_ short and ciliform. All lyrifissures well visible, *ip* located between *p*_1_ and *p*_2_ on left side while it between *p*_2_ and *p*_3_ on right side, *ips* between *h*_3_ and *p*_3_. Opisthonotal gland openings located close to lyrifissure *im.* Vitta marginalis distinct. Lyrifissures *im* and setae *lm* almost located at same level.

***Epimeral and lateral podosomal regions*.** Pedotecta II disproportionately dilated, with smaller anterior parts and larger posterior parts projecting lateroposteriad. Epimeral border I well visible. Apodemes I, II and sejugal apodeme well developed, epimeral foramen present. Sternal apodeme well developed. Epimeral setal formula 3-1-3-3. Epimeral setae slightly barbed, seta *4a* inserted between *4b* and *4c*, and closer to *4c*. Postpodosomal ornamentation present.

***Anogenital region*.** Genital plates relatively smooth. Four pairs of genital setae (mutual distances *g*_1_–*g*_1_≈*g*_2_–*g*_2_≈*g*_4_–*g*_4_<*g*_3_–*g*_3_, *g*_2_ longer than the rest). Aggenital lyrifissures located close and anterolateral to genital aperture. A pair of ventral groove present between genital aperture and ventral ridge. One pair of aggenital, two pairs of anal (mutual distances *an*_1_–*an*_1_<*an*_2_–*an*_2_) and three pairs of adanal setae short, similar in length. Setae *ad*_3_–*ad*_3_ below level of anterior margin of anal opening. Adanal lyrifissures located in diagonal position and close to anal aperture, below level of anterior margin of anal opening.

***Gnathosoma*.** Subcapitular setae barbed. Rutellum pantelobasic, with typical dentation and rutellar brush. Chelicera chelate-dentate; with a minute denticle proximal to seta *cha*; *cha* longer than *chb*; Trägårdh’s organ narrowly triangular. Palp with usual setal formula: 0-2-1-3-8 (+**ω**); setae of trochanter to tibia barbed. Tarsus with four short, blunt distal eupathidia–*acm*, *su*, (*ul*); base of solenidion **ω** constrainted by surface of tarsus, and thus adjacent to setae *ul*’, *ul*” medioanteriorly. Postpalpal setae erect, smooth.

***Legs*.** Monodactylous. Claw of each leg strong and smooth. Formulae of leg setation and solenidia (Table 3): I (1-4-3-4-16) [1-2-2], II (1-4-3-3-15) [1-1-2], III (1-2-1-2-15) [1-1-0], IV (1-2-2-2-12) [0-1-0]. Leg setae *u* setiform (L-type) on tarsi I, thorn-like (S-type) on tarsi II–IV.

#### Material examined.

***Holotype***: female (in alcohol, ZLH-12-276): China, Yunnan Province, Yingjiang County, Taiping Town, Huilonghe Reservoir, 24°40'20"N, 97°45'28"E, 1769 m a. s. l., litter and soil under moss, 24 October 2012, collected by Lihao Zheng.

#### Type deposition.

Type specimen is deposited in the collection of the IZAS.

#### Etymology.

The specific name “*sinutus*” is from Latin for “sinus” refers to the ventral groove between genital aperture and ventral ridge.

#### Remarks.

As possessing the conspicuously developed costula and the distinctly dilated posterior pedotecta II, which are diagnostic characters of the genus, this new species should be placed into the genus *Eurostocepheus*. The new species can be easily distinguished from other known species of this genus by its huge body size, eight pairs of notogastral setae and its ventral groove between the genital aperture and the ventral ridge.

### ﻿ Eurostocepheus (Eurostocepheus) aquilinus

Taxon classificationAnimaliaSarcoptiformesOtocepheidae

Aoki, 1965

A31600F4-A5FB-5162-9233-6038C83FD42D

**Figure 9.**
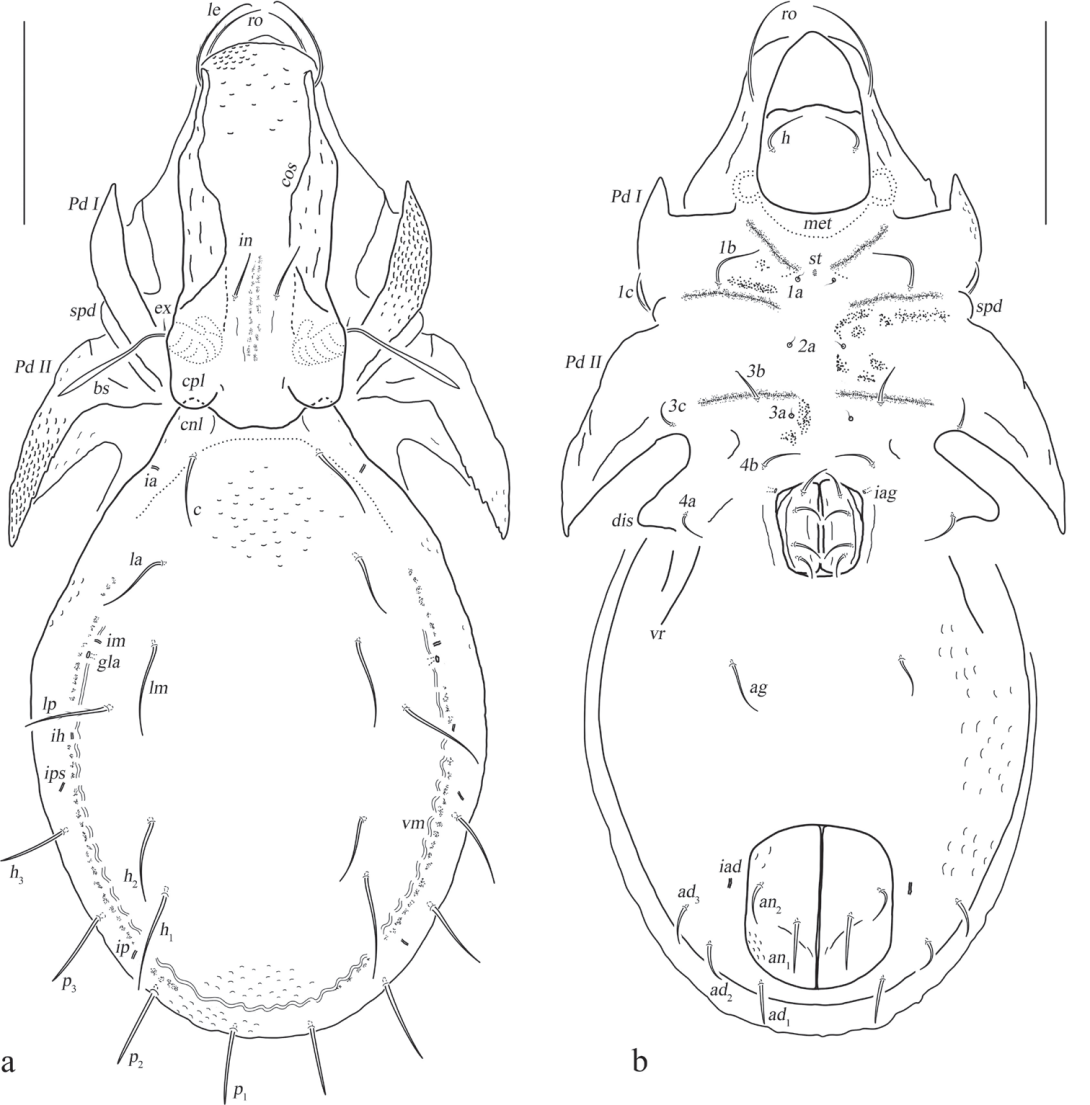
Eurostocepheus (Eurostocepheus) aquilinus Aoki, 1965, adult: **a** dorsal aspect (legs removed) **b** ventral aspect (legs and mouthparts removed). Abbreviations and notations explained in text. Scale bars: 200 µm.

**Figure 10.**
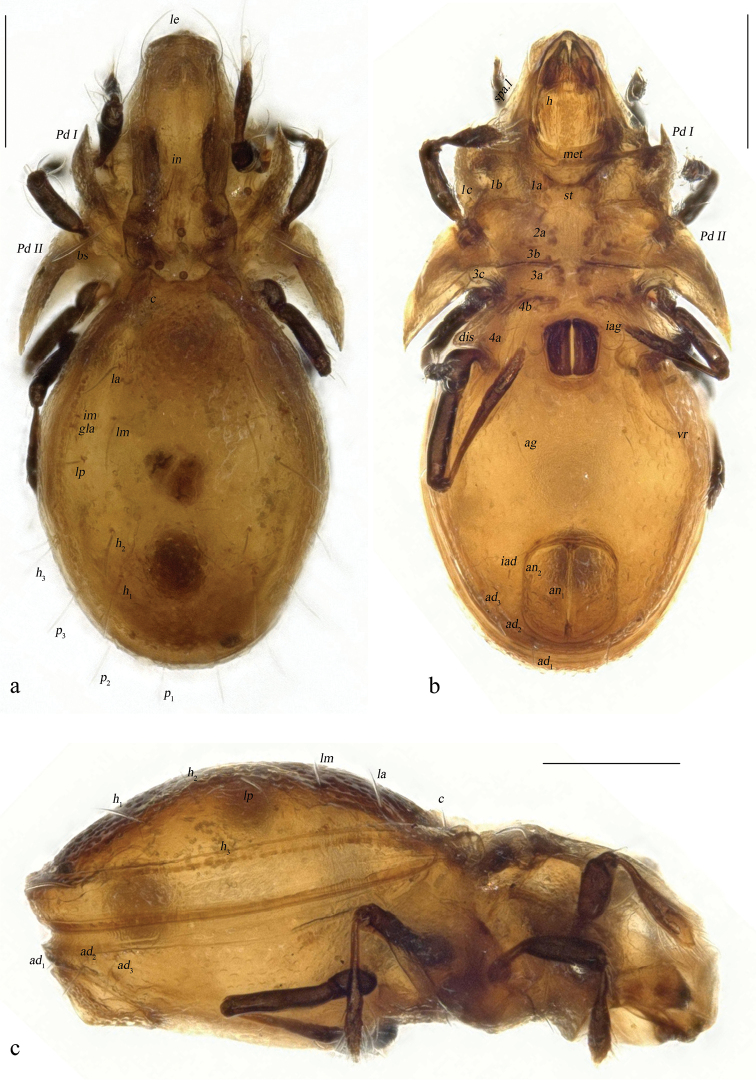
Eurostocepheus (Eurostocepheus) aquilinus Aoki, 1965, adult, microscope images: **a** dorsal view **b** ventral view **c** lateral view. Abbreviations and notations explained in text. Scale bars: 200 µm.

New record for China Figures 9 , 10

Eurostocepheus aquilinus Aoki, 1965: 334–339, figs 142–146; Ermilov, Niedbała and Anichkin 2012: 23.

#### Diagnosis.

Body size: 1000 × 510. Body ratio (length/width): 2.0. Costula strong, thin anteriorly, and thick posteriorly. Lamelliform expansion sigmoid and passing between base of setae *ro* and *le* in lateral view. Ten pairs of notogastral setae nearly equal in length. Sternal apodeme short and rounded. Epimeral setal formula 3-1-3-2. Epimeral setae slightly barbed, seta *1a*, *2a*, *3a* short and thin, hardly visible.

#### Description.

***Measurements*** (ZLH-20-029, male). Body length: 1000, body width: 510. Setae length and mutual distance: *ro* 130, *le* 140, *bs* 150, *in* 60, *ex* 20; notogastral setae range 70–110. Mutual distance: *c*–*c* 140, *la*–*la* 190, *lm*–*lm* 210, *lp*–*lp* 310, *h*_2_–*h*_2_ 220, *h*_1_–*h*_1_ 210.

***Integument*.** Body color dark brownish. Body surface covered with foveola.

***Prodorsum*.** Rostrum rounded. Both rostral setae and lamellar setae curved inward, slightly barbed outside. Lamellar setae inserted behind tip of costula. Interlamellar setae slightly barbed. Bothridial setae with a long fusiform head and a curved peduncle. Exobothridial setae short, hard to see in dorsal view. Costula strong, thin anteriorly, and thick posteriorly. Bothridium opening laterally, dorsal bothridial plate nearly straight, ventral bothridial plate invisible in dorsal view. Tutorium fainted. Lamelliform expansion sigmoid and passing between bases of setae *ro* and *le* in lateral view. Lateral prodorsal condyles broadly rounded, median prodorsal condyles absent. Mutual distance between ventral bothridial plate nearly equal with that between lateral prodorsal condyles.

***Notogaster*.** L/W of notogaster about 1.3. Lateral notogastral condyles triangular. Median notogastral condyles absent. Ten pairs of notogastral setae nearly equal in length. All lyrifissures well visible, *ip* located between *p*_2_ and *p*_3_, *ips* between *h*_3_ and *p*_3_. Opisthonotal gland openings located close to lyrifissure *im.* Vitta marginalis distinct. Lyrifissures *im* and setae *lm* located nearly same level.

***Epimeral and lateral podosomal regions*.** Epimere I with distinct, long ridge extend to pedotectum I. Apodemes I, II and sejugal apodeme well developed. Sternal apodeme short and rounded. Epimeral setal formula 3-1-3-2. Epimeral setae slightly barbed, seta *1a*, *2a*, *3a* short and thin, hardly visible.

***Anogenital region*.** Genital plates sculptured irregularly with several strong furrows. Four pairs of genital setae (largest mutual distance is *g*_3_–*g*_3_). Aggenital lyrifissures located close and anterolateral to genital aperture. One pair of aggenital, two pairs of anal (mutual distances *an*_1_–*an*_1_<*an*_2_–*an*_2_) and three pairs of adanal setae similar in length. Setae *ad*_3_–*ad*_3_ below level of anterior margin of anal opening. Adanal lyrifissures located longitudinally and close to anal aperture, below level of anterior margin of anal opening.

***Gnathosoma*.** Subcapitular setae barbed. Rutellum pantelobasic, with typical dentation and rutellar brush. Chelicera chelate-dentate; with a minute denticle proximal to seta *cha*; *cha* longer than *chb*; Trägårdh’s organ narrowly triangular. Palp with usual setal formula: 0-2-1-3-8 (+**ω**); setae of trochanter to tibia barbed. Tarsus with four short, blunt distal eupathidia–*acm*, *su*, (*ul*); base solenidion **ω** constrainted by surface of tarsus, and thus adjacent to setae *ul*’, *ul*” medioanteriorly. Postpalpal setae erect, smooth.

***Legs*.** Monodactylous. Claw of each leg strong and smooth. Formulae of leg setation and solenidia (Table 2): I (1-4-3-4-16) [1-2-2], II (1-4-3-3-15) [1-1-2], III (1-2-1-2-15) [1-1-0], IV (0-2-2-2-12) [0-1-0]. Leg setae *u* setiform (L-type) on tarsi I, thorn-like (S-type) on tarsi II–IV.

**Table 2.** Leg setation and solenidia of adult Eurostocepheus (Eurostocepheus) aquilinus Aoki, 1965.

Leg	Tr	Fe	Ge	Ti	Ta
I	*v*’	*d*, (*l*), *bv*”	(*l*), *v*’, *σ*	(*l*), (*v*), *φ_1_*, *φ_2_*	(*ft*), (*tc*), (*it*), (*p*), (*u*), (*a*), *s*, (*pv*), *ε*, *ω_1_*, *ω_2_*
II	*v*’	*d*, (*l*), *bv*”	(*l*), *v*’, *σ*	*l*’, (*v*), *φ*	(*ft*), (*tc*), (*it*), (*p*), (*u*), (*a*), *s*, (*pv*), *ω_1_*, *ω_2_*
III	*v*’	*d*, *l*’, e*v*’	*l*’, *σ*	(*v*), *φ*	(*ft*), (*tc*), (*it*), (*p*), (*u*), (*a*), *s*, (*pv*)
IV	-	*d*, e*v*’	*d*, *l*’	(*v*), *φ*	*ft*”, (*tc*), (*p*), (*u*), (*a*), *s*, (*pv*)

#### Material examined.

One male (in alcohol, ZLH-20-029): China, Yunnan Province, Ruili County, Nongdao Town, Nankaiba, 23°55'49"N, 97°32'7"E, 752 m a. s. l., litter and soil under fern, 25 May 2020, collected by Lihao Zheng.

#### Specimen deposition.

Specimen is deposited in the collection of the IZAS.

#### Remarks.

The specimen checked in this study is almost coincident (shape of lamelliform expansion, different kind of epimeral setae in shape, etc.) with the original description given by Aoki. Here, we provide a supplementary description of this species with new figures and information about morphological characters of this species.

### ﻿ Eurostocepheus (Eurostocepheus) mahunkai

Taxon classificationAnimaliaSarcoptiformesOtocepheidae

Mondal & Kundu, 1999

9FD39D0E-6F66-5588-B433-87498A4F3051

**Figure 11.**
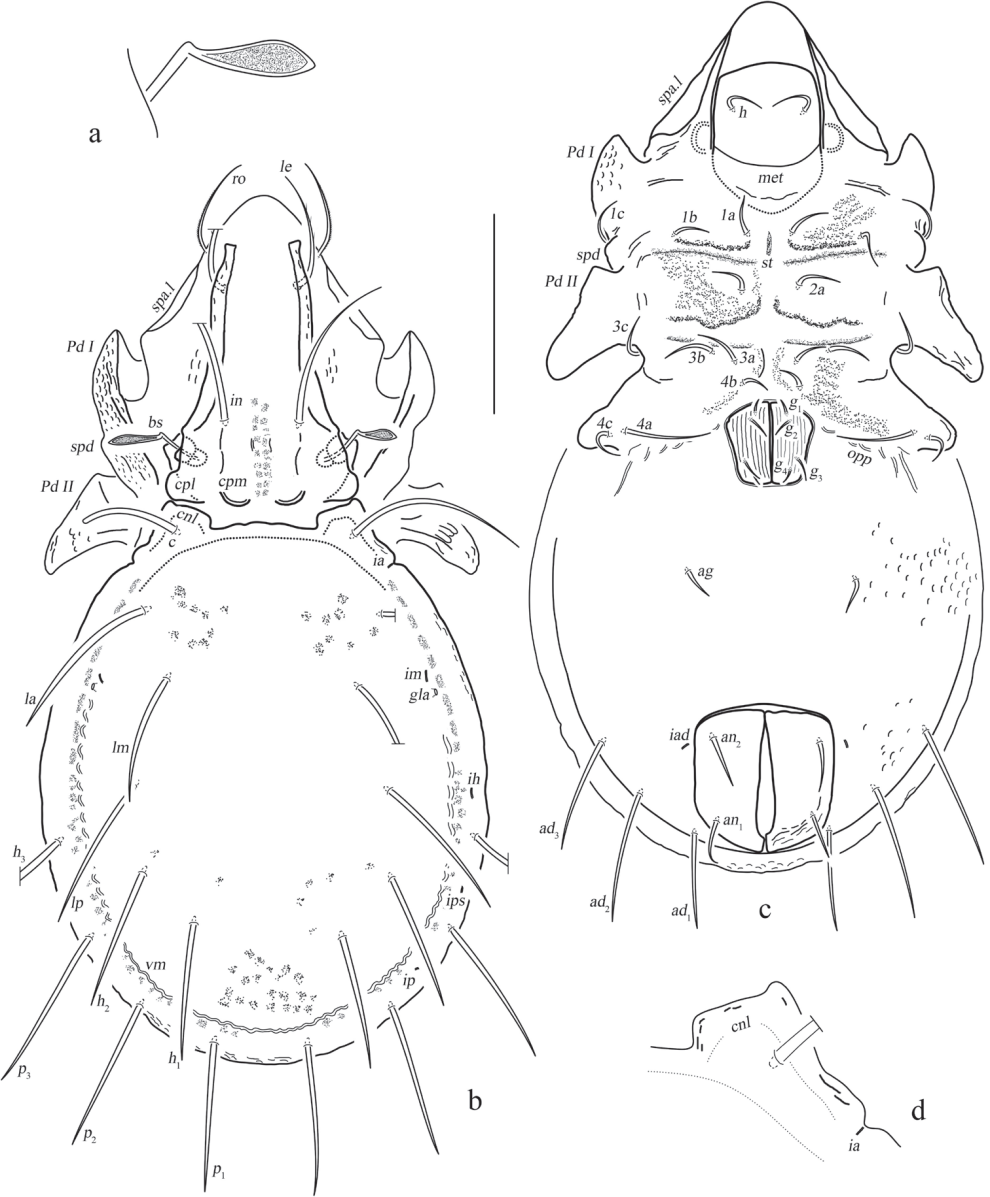
Eurostocepheus (Eurostocepheus) mahunkai Mondal & Kundu, 1999, adult: **a** bothridial seta **b** dorsal aspect (legs removed) **c** ventral aspect (legs and mouthparts removed) **d** lateral notogastral condyle (right). Abbreviations and notations explained in text. Scale bars: 200 µm.

**Figure 12.**
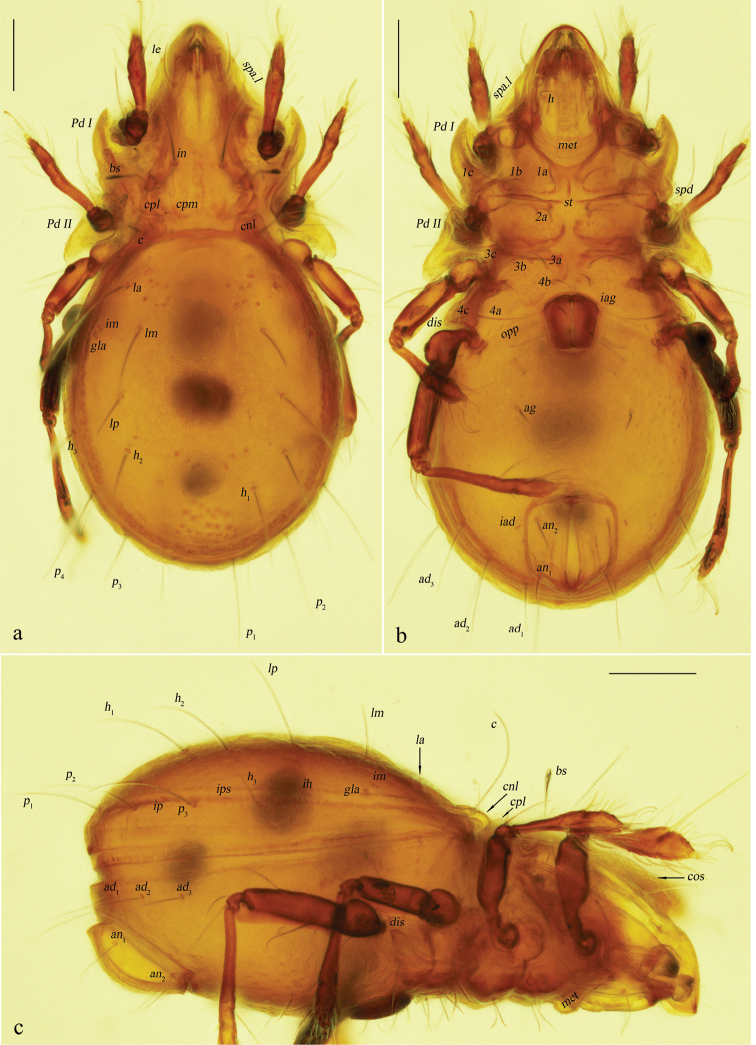
Eurostocepheus (Eurostocepheus) mahunkai Mondal & Kundu, 1999, adult, microscope images: **a** dorsal view, ventral view **c** lateral view. Abbreviations and notations explained in text. Scale bars: 100 µm.

**Figure 13.**
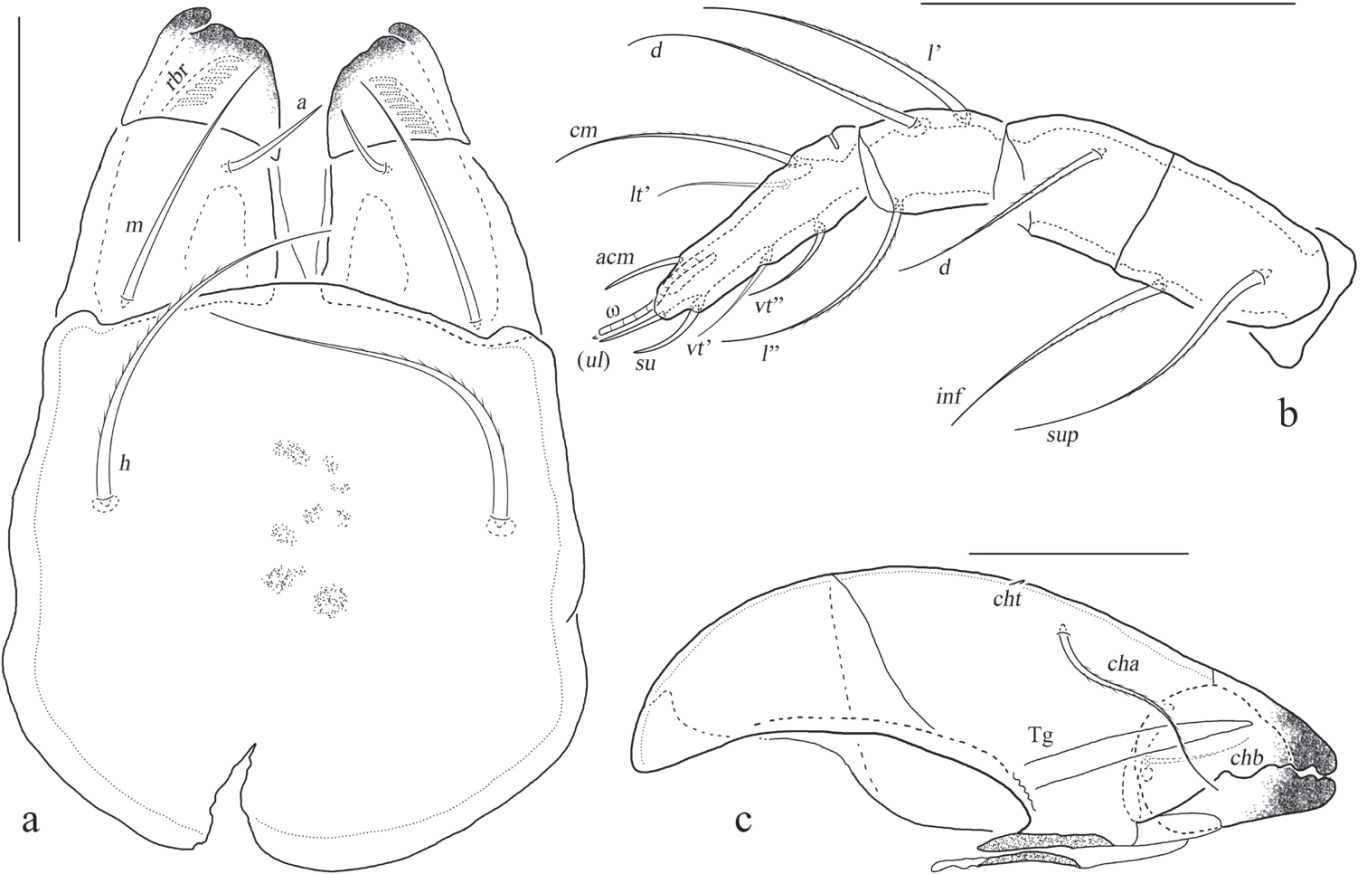
Eurostocepheus (Eurostocepheus) mahunkai Mondal & Kundu, 1999, adult: **a** subcapitulum, ventral view **b** left palp, abaxial view **c** left chelicera, adaxial view. Scale bars: 50 μm.

**Figure 14.**
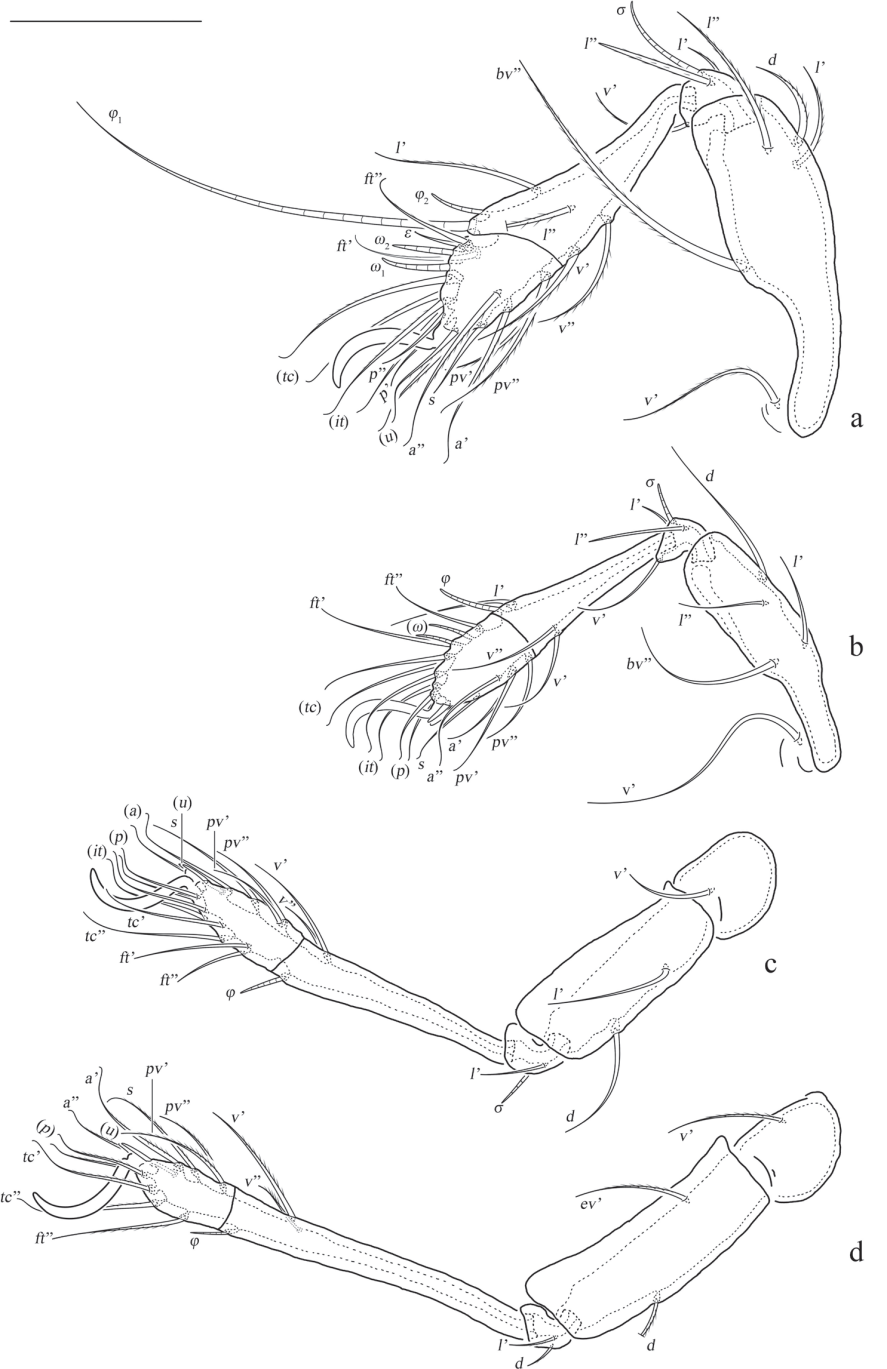
Eurostocepheus (Eurostocepheus) mahunkai Mondal & Kundu, 1999, adult: **a** subcapitulum, ventral view **b** left palp, abaxial view **c** left chelicera, adaxial view. Scale bars: 50 μm.

New record for China Figures 11 , 12 , 13 , 14

Eurostocepheus (Eurostocepheus) mahunkai Mondal & Kundu, 1999: 180–186, figs 1–16.

#### Diagnosis.

Body size: 830 × 420. Body ratio (length/width): 2.0. Mutual distance of costula gradually narrow from base to tip. Two pairs of prodorsal condyles present, lateral prodorsal condyles broadly rounded, with bottom straight, median prodorsal condyles rounded, not conspicuous, well separated from each other. Lateral notogastral condyles trapezoid, with triangular tip outside. Epimeral setal formula 3-1-3-3. Genital plates with longitudinal furrows.

#### Description.

***Measurements*** (holotype, male). Body length: 830, body width: 420. Setae length and mutual distance: *ro* 120, *le* 130, *bs* 100, *in* 160, *ex* 10; notogastral setae range 150–190; *c*–*c* 170, *la*–*la* 220, *lm*–*lm* 180, *lp*–*lp* 230, *h*_2_–*h*_2_ 240, *h*_1_–*h*_1_ 130.

***Integument*.** Body color light brownish. Body surface densely foveolate (not well visible on notogaster).

***Prodorsum*.** Rostrum broadly rounded. Rostral setae moderately curved inward, densely barbed outside. Lamellar setae inserted behind tip of costula, curved inward, roughened externally. Interlamellar setae slightly barbed. Bothridial setae with a long fusiform head and a strongly curved peduncle. Exobothridial setae short. Mutual distance of costula gradually narrow from base to tip. Bothridium opening laterally, dorsal bothridial plate nearly straight, ventral bothridial plate triangular in dorsal view. Tutorium fainted. Two pairs of prodorsal condyles present, lateral prodorsal condyles broadly rounded, with bottom straight, median prodorsal condyles rounded, not conspicuous, well separated from each other. Mutual distance between ventral bothridial plate nearly equal with that between lateral prodorsal condyles. Subpedotectum well developed.

***Notogaster*.** L/W of notogaster about 1.2. Surface of notogaster relatively smooth in dorsal view. Anterior margin of notogaster slightly curved forward. Lateral notogastral condyles trapezoid, with triangular tip outside. Median notogastral condyles absent. Ten pairs of notogastral setae slightly barbed, setiform. A pair of notchs present in external margin of anterior notogaster, beside lyrifissure *ia.* All lyrifissures well visible, *ip* located between *p*_2_ and *p*_3_, *ips* between *h*_3_ and *p*_3_. Opisthonotal gland openings located posterior and close to lyrifissure *im.* Vitta marginalis distinct. Lyrifissures *im* and setae *lm* located nearly same line.

***Epimeral and lateral podosomal regions*.** Epimere I with distinct, long, transverse ridge. Apodemes I, II and sejugal apodeme well developed, apodeme III short. Sternal apodeme well developed. Epimeral setal formula 3-1-3-3. Seta *4a* inserted between *4b* and *4c*, and closer to *4c*. Postpodosomal ornamentation present.

***Anogenital region*.** Genital plates covered with longitudinal furrows. Four pairs of genital setae (mutual distances *g*_1_–*g*_1_≈*g*_2_–*g*_2_≈*g*_4_–*g*_4_<*g*_3_–*g*_3_). Aggenital lyrifissures located close and anterolateral to genital aperture. One pair of aggenital, two pairs of anal (mutual distances *an*_1_–*an*_1_≈*an*_2_–*an*_2_) and three pairs of adanal setae similar in length. Setae *ad*_3_–*ad*_3_ below level of anterior margin of anal opening. Adanal lyrifissures located in diagonal position and close to anal aperture, below level of anterior margin of anal opening.

***Gnathosoma*.** Subcapitular setae barbed, flagelliform at tips. Rutellum pantelobasic, with typical dentation and rutellar brush. Chelicera chelate-dentate; with a minute denticle proximal to seta *cha*; *cha* longer than *chb*, both of them barbed; Trägårdh’s organ narrowly triangular. Palp with usual setal formula: 0-2-1-3-8 (+**ω**); setae of trochanter to tibia barbed. Tarsus with four short, blunt distal eupathidia–*acm*, *su*, (*ul*); other tarsal setae smooth or with sparse, inconspicuous barbs; base of solenidion **ω** constrainted by surface of tarsus, and thus adjacent to setae *ul*’, *ul*” medioanteriorly. Postpalpal setae erect, smooth.

***Legs*.** Monodactylous. Claw of each leg strong and smooth. Formulae of leg setation and solenidia (Table 3): I (1-4-3-4-16) [1-2-2], II (1-4-3-3-15) [1-1-2], III (1-2-1-2-15) [1-1-0], IV (1-2-2-2-12) [0-1-0]. Leg setae *u* setiform (L-type) on tarsi I, thorn-like (S-type) on tarsi II–IV.

**Table 3.** Leg setation and solenidia of adult Eurostocepheus (Eurostocepheus) sinutus sp. nov. and Eurostocepheus (Eurostocepheus) mahunkai Mondal & Kundu, 1999.

Leg	Tr	Fe	Ge	Ti	Ta
I	*v*’	*d*, (*l*), *bv*”	(*l*), *v*’, *σ*	(*l*), (*v*), *φ_1_*, *φ_2_*	(*ft*), (*tc*), (*it*), (*p*), (*u*), (*a*), *s*, (*pv*), *ε*, *ω_1_*, *ω_2_*
II	*v*’	*d*, (*l*), *bv*”	(*l*), *v*’, *σ*	*l*’, (*v*), *φ*	(*ft*), (*tc*), (*it*), (*p*), (*u*), (*a*), *s*, (*pv*), *ω_1_*, *ω_2_*
III	*v*’	*d*, *l*’, e*v*’	*l*’, *σ*	(*v*), *φ*	(*ft*), (*tc*), (*it*), (*p*), (*u*), (*a*), *s*, (*pv*)
IV	*v*’	*d*, e*v*’	*d*, *l*’	(*v*), *φ*	*ft*”, (*tc*), (*p*), (*u*), (*a*), *s*, (*pv*)

#### Material examined.

One male (in alcohol, ZLH-12-229): China, Yunnan Province, Ruili City, Nongdao Town, 23°59'49.1"N, 97°39'10.79"E, 1150 m a. s. l., primary forest, litter and soil under bamboo, 24 October 2012, collected by Lihao Zheng.

#### Specimen deposition.

Specimen is deposited in the collection of the IZAS.

#### Remarks.

The specimen collected from Yunnan, Southwest China is morphologically coincident (shape of costula, unsmooth genital plates, epimeral setal formula, etc) with E. (E.) mahunkai described and illustrated by Mondal and Kundu (1999) from Darjeeling, Bengal, India, except for the shape of lateral notogastral condyles (trapezoid in our specimen vs. triangular in Mondal and Kundu’s description). Considering that there is intraspecific variation in the shape of notogastral or prodorsal condyles in Otocepheidae (Aoki 1967; Zheng and Chen 2020), we identified this specimen as E. (E.) mahunkai, which has not been recorded in China before.

## Supplementary Material

XML Treatment for Basiceramerus ovatus

XML Treatment for Eurostocepheus (Eurostocepheus) sinutus

XML Treatment for Eurostocepheus (Eurostocepheus) aquilinus

XML Treatment for Eurostocepheus (Eurostocepheus) mahunkai
